# Cumulative effects of unemployment on health in midlife: the buffering effect of social participation

**DOI:** 10.3389/fpubh.2025.1611358

**Published:** 2025-08-12

**Authors:** Xiaoqun Chen, Tsz Lam Lau, Kerui Hu, Jiangjun Yuan

**Affiliations:** ^1^Hangzhou Vocational and Technical College, Hangzhou, China; ^2^Columbia University, New York City, NY, United States

**Keywords:** unemployment, health effects, social participation, gender, middle-aged and older adults

## Abstract

**Objective:**

The purpose of this study was to investigate the cumulative impact of unemployment on the overall health of middle-aged and older adults in China, and to explore the moderating role of social participation and its gender differences.

**Methods:**

This study utilized panel data from the China Health and Retirement Longitudinal Study (CHARLS). The sample included 5,973 individuals (2,830 men and 3,143 women), with respondents categorized by employment status and unemployment duration. Statistical methods included fixed effect model and factor analysis.

**Results:**

(1) Unemployment significantly damages health, with more pronounced adverse effects among men than women. (2) For men, longer unemployment durations result in progressively worse health outcomes, with the greatest negative impact observed at 6–8 years of unemployment. (3) Women experience significant health declines during short-term (0–2 years) and medium-term (3–5 years) unemployment only, with no additional deterioration in long-term unemployment. (4) Informal social participation significantly buffers the negative health effects of unemployment, particularly for men, while formal social participation shows no protective effect.

**Conclusion:**

(1) This study demonstrates the cumulative and gendered impact of unemployment on the health of middle-aged and older adults in China. (2) Informal social participation provides a meaningful buffer against the adverse health consequences of unemployment, particularly for men, whereas formal social activities may not offer such protection and may even have negative effects in certain scenarios. (3) These findings underscore the importance of strengthening informal social support networks to mitigate health risks associated with job loss among older populations.

## 1 Introduction

In middle and old age, people are more likely to experience life events that trigger role changes and increase psychological stress (1). Unemployment defined as the involuntary loss of paid employment (2), is a common trigger for such role transitions among middle-aged and older adults (3) and can increase susceptibility to disease (4). Compared with developed countries, the welfare support for the unemployed in China is less comprehensive, rendering unemployment more detrimental to health (5). According to China's seventh national population census conducted in 2020, 27.49% of people aged 65–69 were still working, and in rural areas, as many as 46.11% of people aged 60–64 remained employed (6). This phenomenon is largely attributed to two main factors: insufficient pension payments that fail to cover basic living expenses, and the desire of some older adults to maintain social purpose and self-worth beyond the official retirement age. Consequently, unemployment in older adults is a significant issue in China. Losing a job leads to altered or lost social roles, which in turn adversely affects health (7). In addition, middle-aged and older individuals often face greater challenges in securing new employment, as their skills may not easily transfer to other roles, resulting in prolonged periods of unemployment compared to younger workers (8).

Previous research broadly categorizes the negative impacts of unemployment into three areas. First, unemployment results in financial stress due to the loss of income and ensuing economic insecurity (9). Second, unemployment has been linked to various physical health issues, including diabetes (10), hypertension, coronary heart disease, and other chronic diseases (11, 12). Third, unemployment harms mental health, and unemployment is associated with higher levels of depression (13), suicidal tendencies (14), and anxiety (15). Some studies, however, found no significant relationship between unemployment and certain chronic conditions (16, 17). Some scholars have also stated that the impact of unemployment on health depends on the length of unemployment (18) and the gender of the unemployed people (19). Therefore, the inconsistent findings regarding the impact of unemployment on health may be attributed to a failure to take into account both the duration of unemployment and the gender of the unemployed. Classen et al. (14) found that long-term unemployed individuals, particularly those approaching 26 weeks of unemployment, face an increased risk of suicide, whereas individuals who are newly unemployed show no significant rise in suicide risk. Acevedo et al. (18) reported that both short-term and long-term unemployment elevate the risks of anxiety, depression, and poor self-perceived health for both men and women, though the risks are notably higher for those who are long-term unemployed. They also observed that the negative health effects of unemployment are relatively smaller among older women. Strandh et al. (19) discovered that in Ireland, where a traditional male breadwinner and female homemaker model prevails, male unemployment results in greater economic and psychological harm. In contrast, in Sweden, a country with a high level of gender equality, the psychological impact of unemployment does not significantly differ between men and women.

Social participation can affect the health level of middle-aged and older adults in direct or buffering ways. Regarding the direct effect, according to the activity theory of aging, older adults who actively engage in social and daily activities are better able to maintain their sense of identity, social roles, psychological satisfaction, and overall life satisfaction, thus achieving successful aging (20). The buffering effect is somewhat more complex. Social participation can provide older adults with social support, which refers to the emotional, informational, or practical assistance received from friends, family, or colleagues (21). Social support can play a “buffering” role in the face of stressful events, helping to mitigate the negative impacts of stress on both psychological and physical health (22, 23). In other words, when individuals encounter difficulties, social support enables them to cope more effectively and reduce harm. Therefore, we propose that social participation can also buffer the negative effects of stressful events on the mental and physical health of older adults. However, scholarly research on the relationship between social participation and health has primarily focused on its direct (main) effects; for example, some scholars have explored the impact of volunteering on life satisfaction (7), while others have directly examined the effect of social participation on depression levels among older adults (20). Since social participation can provide social support, and social support is known to buffer the negative health impacts of stress, most current studies focus on the buffering role of social support while overlooking the potential buffering role of social participation itself. This is why we aim to directly investigate how social participation may buffer the health impacts of stress. In addition, previous studies have categorized social participation into two types: informal and formal. Informal social participation mainly refers to interactions with family, friends, and neighbors, while formal social participation refers to activities organized by institutions with the goal of accomplishing specific tasks. Research has found that informal social participation, due to its greater focus on emotional bonds and close interactions, may have a stronger effect in preventing depression and improving life satisfaction among middle-aged and older adults (20, 24).

Based on the above analysis, we mainly put forward the following five hypotheses:

- H1: Unemployment has a negative impact on health among middle-aged and older adults.
- H2: The detrimental effect of unemployment on health increases with longer durations of unemployment (cumulative effect).
- H3: The negative health impact of unemployment is more severe for men than for women under Chinese context.
- H4: Social participation moderates the relationship between unemployment and health by buffering the adverse effect of unemployment.
- H5: Informal social participation provides a stronger buffering effect on health than formal social participation.

In summary, previous studies exhibit methodological limitations and content deficiencies when examining the relationship between unemployment and health. For methodological limitations, most studies rely on cross-sectional data that do not adequately control for unobserved individual characteristics, resulting in potential omitted variable bias (25). In terms of deficiencies, first of all, many studies ignore the dynamics of unemployment, that is, the length of unemployment, when analyzing the impact of unemployment on health. Secondly, regarding the selection of health indicators, which are basically mental health (23, 26, 27), few studies have explored the impact of unemployment on overall health. Finally, while some research has highlighted the moderating role of social support in the relationship between job loss and health (23). Considering social participation is an important source of social support (28), fewer studies have investigated how social participation itself may buffer the adverse health effects of job loss. This paper addresses these gaps by focusing on the Chinese context and examining the moderating effects of social participation, with special attention to gender differences. Based on the above analysis, the main research objectives of this study are to: (1) Describe gender differences in unemployment patterns among middle-aged and older adults. (2) Investigate the relationship between unemployment (and its duration) and overall health. (3) Examine how social participation moderates the impact of unemployment on health in middle-aged and older adults.

## 2 Methods

### 2.1 Data source

This study utilizes data from the China Health and Retirement Longitudinal Study (CHARLS), which was conducted by Peking University using a multistage sampling strategy. Interviews were carried out in 2011 (baseline), 2013, 2015, and 2018. Given that work status data prior to 2011 were unavailable, only respondents employed at baseline were included in analyses related to unemployment duration. To ensure data quality, respondents who underwent retirement procedures (or early retirement) or had missing values for key variables, as well as those over 80 years old, were excluded. We ultimately obtained a sample set containing 5,973 respondents. Moreover, we compared the demographic characteristics of the 5,973 respondents included in our final analytic sample with those who were excluded and found no significant differences. The CHARLS survey was conducted by Peking University with ethical approval (IRB00001052–11015), and all participants provided informed consent. This study uses de-identified secondary data, and no additional ethical approval was required.

### 2.2 Measures

#### 2.2.1 Dependent variables

Health was measured using several indicators, including Instrumental Activities of Daily Living, Basic Activities of Daily Living, depression score, pain situation, and episodic memory. These multiple indicators have overlapping parts and independent parts. This study conducted a factor analysis on these indicators and obtained three common factors: physical health, psychological health, and cognitive health. The overall health score was constructed using weighted contributions from these three factors. The score to measure the overall health of the middle-aged and older adults is computed as follows:


$$\begin{array}{l} health\;score=\alpha_{1}\;*\;physical\;health+\alpha_{2}\;*\;psychological\;health\\ \;+\alpha_{3}\;*\;cognitive\;health\; \end{array}$$

where α_1_, α_2_, and α_3_ represent the weight of common factors in the total score. The weight is determined by the contribution rate of each factor in the cumulative variance. In the factor analysis in this study, the weights of the three factors are finally obtained as 0.53, 0.25, and 0.22, respectively, which also indicates that physical health can explain more than half of the changes in health level.

#### 2.2.2 Independent variables

The independent variables of this article are the respondents' working status and changes in working status. Unemployment status was recorded in each wave and classified into three categories: employed, unemployed-but-not-retired, and not working due to retirement. This study defines unemployment as a respondent who is neither working nor retired. In this study, unemployment is operationalized as the state of being not in employment while not retired. Accordingly, a dummy variable was generated (1 = unemployed and not retired; 0 = employed). In order to study the impact of unemployment duration on the health level of the unemployed, this study divided the employment status into four categories by comparing the working status of the previous interviews with the current working status. The four categories are: consistently employed, unemployed for 0–2 years, unemployed for 3–5 years, and unemployed for 6–8 years. “Consistently employed” represents respondents that have a job in the current period and also had a job in the previous period. “Unemployed for 0–2 years” represents respondents that have no job in the current period and had a job in the previous period. “Unemployed for 3–5 years” represents respondents who were unemployed in the current period and the previous period, but were employed before those two periods. “Unemployed for 6–8 years” refers to respondents who had a job in the first period and no job in the next three periods. In addition, this study cannot obtain the length of unemployment of respondents who did not work in the first period because there is no sample data from the previous period. In order to ensure the rigor of the research, this study only retains samples with jobs in the first period.

#### 2.2.3 Moderator variables

There are two moderator variables in this study, namely formal social participation and informal social participation. Previous studies classified social activities into informal and formal based on factors such as intimacy, purpose, and participation intensity (20). Social activities that are purposeful and require a lower level of intimacy among participants are categorized as formal, whereas those that are less goal-oriented and demand a higher degree of closeness among participants are deemed informal. Based on these definitions and the social activities included in the CHARLS questionnaire, formal social activities include “participating in club activities,” “participating in volunteer or charity activities,” “taking regular and unpaid care of patients who do not live with you,” and “participating in training courses or attending school.” Formal social participation is a binary variable (1 = participation in at least one formal activity in the past month; 0 = no participation in the past month). Informal social activities include “going to parks or other places to dance, exercise, practice qigong, etc.,” “socializing with friends” and “playing mahjong, chess, cards, going to community rooms.” This is also coded as a binary variable (1 = participation in at least one informal activity in the past month; 0 = no participation in the past month).

#### 2.2.4 Control variables

Based on previous research (23), this study controlled for several demographic and socioeconomic factors including age, education level, and place of residence. Family structure variables (e.g., marital status, number of living children, history of child loss, and parental survival status) were also included. In addition, two health-related behaviors (smoking and drinking) and financial information (annual income and private insurance status) were controlled to mitigate omitted variable bias.

### 2.3 Econometric model

To address potential endogeneity due to omitted variable bias caused by the individual heterogeneity that does not change with time (e.g., unobserved individual characteristic such as inherent ability or personality), this study employs the individual fixed effect model. In addition, the Hausman test also supports fixed effect model, the model of this study is as follows:

(1) Two-class main effect model (baseline model: working)

This baseline model divides respondents simply into “employed” and “unemployed” categories to estimates the average impact of unemployment on the health level of the respondents. The model is shown in Equation (1):


(1) $$\begin{array}{l} health_{it}=\beta_{0}+\beta_{1}unemployed_{it}+\beta_{2}informal_{it}+\beta_{3}formal_{it}\\ \;+\beta_{4}control_{it}+u_{i}+\varepsilon_{it} \end{array}$$

Where *health*_*it*_ represents overall health score for respondent *i* at time *t*, *unemployed*_*it*_ is a dummy variable, and 1 indicates that respondent *i* was unemployed at time t. *informal*_*it*_ and *formal*_*it*_ indicate whether the respondents participate in informal and formal social activities, respectively. *control*_*it*_ indicates control variables, β_0_−β_4_ are coefficients, where β_1_ represents the average health effect of unemployment, *u*_*i*_ is the fixed effect of individual *i*, which will not change with time. ε_*it*_ is the error term.

(2) Four-class main effect model (Base model: working)

On the basis of the “Two-class main effect model,” this model further distinguished the duration of unemployment of respondents and divided respondents into the following four types: employed, unemployed for 0–2 years, unemployed for 3–5 years, and unemployed for 6–8 years. The model is shown in Equation (2):


(2) $$\begin{array}{l} health_{it}=\beta_{0}+\overset{3}{\underset{j=1}{\sum }}\alpha_{j}unemployed_{jit}+\beta_{2}informal_{it}+\beta_{3}formal_{it}\\ \;+\beta_{4}control_{it}+u_{i}+\varepsilon_{it} \end{array}$$

Where *unemployed*_*jit*_ represents respondent *i*'s *j*-th unemployment variable at time t. The first unemployment variable represents 0–2 years of unemployment, the second is 3–5 years of unemployment, and the third is 6–8 years of unemployment, α_*j*_ represents the coefficient in front of the *j*-th unemployment variable.

(3) Two-class moderating effect model

To examine the buffering (moderating) effect of social participation, interaction terms between unemployment status and the moderator variables (*unemploye*_*d*_*it*_ * *informalit*_ and *unemploye*_*d*_*it*_ * *formalit*_) are added to Model (1). The specific Equation is shown in (3):


(3) $$\begin{array}{l} health_{it}=\beta_{0}+\beta_{1}unemployed_{it}+\beta_{2}informal_{it}+\beta_{3}formal_{it}\\ \;+\beta_{4}control_{it}+\beta_{5}{unemployed_{it}\;*\;informal}_{it}\\ \;+\beta_{6}{unemployed_{it}\;*\;formal}_{it}+u_{i}+\varepsilon_{it} \end{array}$$

(4) Four-class moderating effect model

Extending Model (2), this formulation introduces interaction terms for each unemployment duration category with both moderator variables. The specific Equation is shown in (4):


(4) $$\begin{array}{l} health_{it}=\beta_{0}+\overset{3}{\underset{j=1}{\sum }}\alpha_{j}unemployed_{jit}+\beta_{2}informal_{it}+\beta_{3}formal_{it}\\ \;+\overset{3}{\underset{j=1}{\sum }}\gamma_{j}unemployed_{jit}\;*\;informal_{it}+\overset{3}{\underset{j=1}{\sum }}\pi_{j}unemployed_{jit}\\ \;{*\;formal}_{it}+\beta_{4}{\text{c}ontro\text{l}}_{it}+u_{i}+\varepsilon_{it} \end{array}$$

In Equation (4), *unemployed*_*jit*_ * *informal*_*it*_, and *unemployed*_*jit*_ * *formal*_*it*_ represent the interaction terms of informal social participation and formal social participation with the *j*-th unemployment variable, respectively. γ_*j*_ represents the moderating effect of informal social participation on the relationship between *j*-th unemployment variable and health, and π_*j*_ represents the moderating effect of formal social participation on the relationship between *j*-th unemployment variable and health. The other symbolic interpretations of Equation (4) are the same as those of Equation (2).

## 3 Data analysis results

### 3.1 Descriptive statistics

Table 1 summarizes the characteristics of respondents in 2018. The sample includes 5,973 individuals (2,830 men and 3,143 women). Among them, 4,339 were employed, 953 experienced 0–2 years of unemployment, 430 experienced 3–5 years, and 251 experienced 6–8 years. Notably, there were more men among the consistently employed, whereas more women experienced unemployment of any duration. Most respondents were married, resided in rural areas, did not have private insurance, and had not experienced child loss. Educational attainment and income levels were generally higher among men, who also reported higher rates of smoking and drinking. Regarding social participation, ~59.0% of male respondents and 57.8% female respondents did not participate in any social activities.

**Table 1:** Characteristics of respondents in 2018 (by unemployment status).

**Status**	**Total (*****n*** = **5,973)**		**Employed (*****n*** = **4,339)**		**Unemployed for 0–2 years (*****n*** = **953)**		**Unemployed for 3–5 years (*****n*** = **430)**		**Unemployed for 6–8 years (*****n*** = **251)**	
	**Male (*n* = 2,830)**	**Female (*n* = 3,143)**	**Male (*n* = 2,252)**	**Female (*n* = 2,087)**	**Male (*n* = 354)**	**Female (*n* = 599)**	**Male (*n* = 133)**	**Female (*n* = 297)**	**Male (*n* = 91)**	**Female (*n* = 160)**
**Demographic characteristics**										
Age	64.106	63.375	62.900	62.011	67.466	64.977	70.617	67.226	71.363	67.931
Edu	6.124	3.326	6.319	3.448	5.373	3.215	5.526	3.061	5.077	2.644
Live city	0.114	.108	0.099	0.075	0.167	0.167	0.173	0.189	0.176	0.163
**Family structure**										
Married	0.900	0.815	0.927	0.866	0.814	0.761	0.789	0.643	0.736	0.675
Parent live	0.325	0.285	0.356	0.323	0.234	0.237	0.158	0.165	0.154	0.188
Child num	2.737	2.864	2.639	2.757	3.006	3.007	3.293	3.104	3.286	3.269
Lose child	0.242	0.276	0.229	0.253	0.271	0.319	0.361	0.370	0.297	0.244
**Financial information**										
Private	0.029	0.019	0.032	0.020	0.017	0.017	0.023	0.024	0.022	0.013
Income	7,507	2,659	8,626	2,974	3,696	1,894	2,777	2,110	1,579	2,441
**Health -related behavior**										
Smoke	0.779	0.071	0.773	0.067	0.808	0.085	0.812	0.067	0.769	0.075
Drink	0.551	0.129	0.595	0.148	0.404	0.093	0.346	0.111	0.319	0.063
**Social participation**										
None	0.590	0.578	0.583	0.592	0.593	0.559	0.639	0.522	0.681	0.569
Informal	0.395	0.409	0.400	0.393	0.398	0.431	0.361	0.465	0.308	0.419
Formal	0.051	0.035	0.058	0.039	0.031	0.023	0.015	0.04	0.011	0.013

However, as unemployment duration increased in men, the non-participation rate increased; for women, the non-participation rate first decreased and then increased with longer unemployment. In addition, the study found that informal social participation was consistently more prevalent than formal participation. For respondents experiencing unemployment, women were more likely to participate in informal social activities than men. For respondents who had not experienced unemployment, men were slightly more likely to participate in informal social activities than women.

Table 2 shows the average health scores of respondents by unemployment status. The health level of unemployed men and women was lower than that of employed men and women. Notably, the health status of females was lower than that of males irrespective of employment status. In addition, the descriptive statistics showed that the health of middle-aged and older male and female respondents became worse as the duration of unemployment increased.

**Table 2:** Average health level and unemployment status of respondents.

**Employment status**	**Last period (2018)**			**All periods (2011–2018)**		
	**Total (*****n*** = **5,241)**	**Male (*****n*** = **2,559)**	**Female (*****n*** = **2,682)**	**Total (*****n*** = **16,010)**	**Male (*****n*** = **7,962)**	**Female (*****n*** = **8,048)**
Total	−0.132	0.034	−0.290	−0.037	0.109	−0.182
Employed	−0.025	0.123	−0.192	0.030	0.161	−0.117
Unemployed for 0–2 years	−0.388	−0.283	−0.453	−0.284	−0.169	−0.351
Unemployed for 3–5 years	−0.458	−0.303	−0.531	−0.439	−0.328	−0.496
Unemployed for 6–8 years	−0.626	−0.610	−0.636	−0.626	−0.610	−0.636

### 3.2 Regression results

#### 3.2.1 Health effects of unemployment

Table 3 reports the regression results of the fixed effects model examining the health effects of unemployment. Columns 1, 2, and 3 display the average effects of unemployment on health for different samples (two-class main effect model), respectively. In contrast, Columns 4, 5, and 6 illustrate the effects of different unemployment durations on health for different samples (four-class main effect model). According to the regression results of “two-class main effect model,” unemployment significantly reduces the health levels for both genders (β = −0.166 vs. β = −0.089, both *p* < 0.05). Meanwhile, the “four-class main effect model” reveals that unemployment lasting 0–2 (β = −0.162, *p* < 0.01), 3–5 (β = −0.176, *p* < 0.01), and 6–8 (β = −0.389, *p* < 0.01) years significantly diminishes health among male respondents, whereas for female respondents, significant reductions in health are observed only for unemployment durations of 0–2 (β = −0.108, *p* < 0.01) years and 3–5 (β = −0.129, *p* < 0.01) years. In addition, Supplementary Tables S1–S3 report the fixed effects regression results examining the main effects of unemployment on physical, cognitive, and psychological health separately. Similar to the composite health results, unemployment consistently shows significant negative effects on physical health across genders, weaker but still notable effects on cognitive health, and the least consistent effects on psychological health.

**Table 3:** Main effect of unemployment on health.

**Variables**	**Main effect: Two-class**			**Main effect: Four-class**		
	**Total**	**Male**	**Female**	**Total**	**Male**	**Female**
Unemployed	**−0.118** ^***^	**−0.166** ^***^	**−0.089** ^***^			
	(0.011)	(0.020)	(0.014)			
Unemployed for 0–2 years				**−0.132** ^***^	**−0.162** ^***^	**−0.108** ^***^
				(0.017)	(0.029)	(0.022)
Unemployed for 3–5 years				**−0.157** ^***^	**−0.176** ^***^	**−0.129** ^***^
				(0.030)	(0.047)	(0.038)
Unemployed for 6–8 years				**−0.242** ^***^	**−0.389** ^***^	−0.119^*^
				(0.051)	(0.082)	(0.066)
Informal	**0.038** ^***^	**0.039** ^***^	**0.038** ^***^	**0.024** ^***^	0.019^*^	**0.030** ^**^
	(0.007)	(0.009)	(0.010)	(0.008)	(0.011)	(0.013)
Formal	−0.001	−0.001	−0.002	−0.026	−0.001	**−0.064** ^**^
	(0.014)	(0.016)	(0.024)	(0.017)	(0.019)	(0.031)
Observations	25,276	11,730	13,546	15,960	7,930	8,030
R-squared	0.065	0.059	0.075	0.093	0.082	0.109
Respondents	7,577	3,428	4,149	6,548	3,115	3,433

The table controls for age, income (logarithm), number of chronic diseases, whether they live in the city, marital status, whether their parents are alive, number of living siblings, number of living children, whether they live with their children, whether they have experienced child bereavement, whether they have private insurance, income, whether they smoke, whether they drink, and number of chronic diseases. The standard deviation in parentheses. ^***^p < 0.01, ^**^p < 0.05, ^*^p < 0.1 (the same below). Bold values in regression indicate statistically significant coefficients (*P* value < 0.05).

In addition, we test the significance of the size between the coefficients. For middle-aged and older male respondents, respondents who had been unemployed for 0–2 years had significantly lower health levels than those who remained employed, and those who had been unemployed for 3–5 years had significantly lower health levels than those who had not lost their jobs. The health of respondents who had been unemployed for 6–8 years was significantly lower by 0.389 than that of respondents who had not lost their jobs. In addition, unemployment for 6–8 years was significantly more damaging than unemployment for 0–2 years or 3–5 years among male respondents. Therefore, the longer the unemployment length, the greater the health damage of middle-aged and older men. In contrast, for middle-aged and older women, those who were unemployed for 0–2 and 3–5 years also showed significantly lower health levels compared to those who remained employed. The health status of respondents who have been unemployed for 6–8 years has no significant impact on their health status. Therefore, the impact of job loss on female respondents did not increase with the length of unemployment.

This study not only compared the average impact of unemployment on the health between middle-aged and older men and women, but also compared the health damage of different unemployment duration in middle-aged and older men and women. The results are shown in Table 4. It shows that the average negative health effects of unemployment are significantly greater for men than for women (*p* < 0.05), with the difference being particularly pronounced in the case of 6–8 years of unemployment.

**Table 4:** Gender comparison of unemployment's effect on health.

**Main effect**	**Total**	**Male**	**Female**	**Test statistics**	***p*-Value**
Unemployed	**−0.118** ^***^	**−0.166** ^***^	**−0.089** ^***^	**7.26** ^***^	0.007
Unemployed for 0–2 years	**−0.132** ^***^	**−0.162** ^***^	**−0.108** ^***^	1.46	0.227
Unemployed for 3–5 years	**−0.157** ^***^	**−0.176** ^***^	**−0.129** ^***^	0.34	0.560
Unemployed for 6–8 years	**−0.242** ^***^	**−0.389** ^***^	**–**0.119^*^	**3.91** ^**^	0.048

The asterisk symbols following coefficients indicate different levels of statistical significance: ^***^p < 0.01, ^**^p < 0.05, ^*^p < 0.1. Bold values in regression indicate statistically significant coefficients (*P* value < 0.05).

#### 3.2.2 The moderating effects of social participation

Table 5 presents the moderating effects of social participation, estimated from Models (3) and (4). Informal social participation significantly buffered the average negative health impacts of unemployment for both men and women. Specifically, the interaction term between unemployment and informal social participation is positive and statistically significant for the total sample (β = 0.091, *p* < 0.01), for men (β = 0.145, *p* < 0.01), and for women (β = 0.067, *p* < 0.01). In contrast, the moderating effects of formal social participation were not statistically significant for either gender.

**Table 5:** The moderating effect of social participation.

**Variables**	**Moderating effect: Two-class**			**Moderating effect: Four-class**		
	**Total**	**Male**	**Female**	**Total**	**Male**	**Female**
Unemployed	**−0.161** ^***^	**−0.233** ^***^	**−0.122** ^***^			
	(0.011)	(0.018)	(0.015)			
Unemployed for 0–2 years				**−0.166** ^***^	**−0.208** ^***^	**−0.134** ^***^
				(0.017)	(0.025)	(0.024)
Unemployed for 3–5 years				**−0.205** ^***^	**−0.227** ^***^	**−0.181** ^***^
				(0.028)	(0.040)	(0.040)
Unemployed for 6–8 years				**−0.299** ^***^	**−0.452** ^***^	**−0.148** ^**^
				(0.044)	(0.060)	(0.065)
Unemployed^*^informal	**0.091** ^***^	**0.145** ^***^	**0.067** ^***^			
	(0.014)	(0.023)	(0.019)			
Unemployed^*^formal	0.002	0.001	0.014			
	(0.038)	(0.059)	(0.053)			
Unemployed for 0–2 years^*^informal				**0.081** ^***^	**0.010** ^***^	**0.066** ^**^
				(0.023)	(0.035)	(0.032)
Unemployed for 3–5 years^*^informal				**0.098** ^***^	**0.129** ^**^	0.094^*^
				(0.038)	(0.060)	(0.051)
Unemployed for 6–8 years^*^informal				**0.165** ^***^	**0.263** ^***^	0.073
				(0.063)	(0.097)	(0.087)
Unemployed for 0–2 years^*^formal				−0.108	0.018	**−0.230** ^**^
				(0.068)	(0.087)	(0.108)
Unemployed for 3–5 years^*^formal				0.198^*^	0.289	0.201
				(0.111)	(0.185)	(0.145)
Unemployed for 6–8 years^*^formal				−0.586^*^	**−1.062** ^***^	−0.113
				(0.301)	(0.393)	(0.458)
Informal	**0.017** ^**^	0.018^*^	0.019^*^	0.009	0.004	0.015
	(0.007)	(0.010)	(0.011)	(0.009)	(0.012)	(0.014)
Formal	−0.001	−0.0003	−0.006	−0.021	3.50e-05	−0.056^*^
	(0.016)	(0.019)	(0.029)	(0.019)	(0.022)	(0.034)
Observations	25,276	11,730	13,546	15,960	7,930	8,030
R-squared	0.068	0.064	0.076	0.096	0.087	0.111
Respondents	7,577	3,428	4,149	6,548	3,115	3,433

The asterisk symbols following coefficients indicate different levels of statistical significance: ^***^p < 0.01, ^**^p < 0.05, ^*^p < 0.1. Bold values in regression indicate statistically significant coefficients (*P* value < 0.05).

When analyzing the moderating effects by unemployment duration, we find that for middle-aged and older men, informal social participation significantly mitigates the health damage associated with all three durations of unemployment: 0–2 years (β = 0.010, *p* < 0.01), 3–5 years (β = 0.129, *p* < 0.05), and 6–8 years (β = 0.263, *p* < 0.01). Moreover, the buffering effect of informal social participation becomes stronger as the duration of unemployment increases. The coefficient for the interaction term rises from 0.010 (0–2 years) to 0.129 (3–5 years) and further to 0.263 (6–8 years), all statistically significant, suggesting a cumulative moderating benefit with prolonged unemployment. Interestingly, for men who were unemployed for 6–8 years, participation in formal social activities exacerbated the negative health impact of unemployment (β = −1.062, *p* < 0.001), indicating a potential adverse effect of formal participation in this context. For middle-aged and older women, only those who were unemployed for 0–2 years experienced significant buffering effects from informal social participation (β = 0.066, *p* < 0.01). The interaction terms for longer durations of unemployment were not significant. Furthermore, formal social participation not only failed to moderate the health effects of unemployment for women, but in the case of 0–2 years of unemployment, it significantly worsened health outcomes (β = −0.230, *p* < 0.01).

In order to compare the gender differences in the moderating effects of social participation on the relationship between unemployment and health, the Wald Test (29) was used in this study to examine the differences in the moderating coefficients between male and female subsamples, and the results are shown in Table 6. In terms of the average health effects of unemployment, this study found that the moderating effect of informal social participation was significantly greater for men than for women (χ^2^ = 3.96, *p* < 0.05). However, the moderating effect of formal social participation was not significant for either gender, and there was no significant gender difference in this regard.

**Table 6:** Gender Comparison of moderating effect of social participation.

**Moderating effect**	**Total**	**Male**	**Female**	**Comparison**	
	**Coefficients**	**Coefficients**	**Coefficients**	**Test statistics (**χ^**2**^**)**	***p*** **-Value**
Unemployed^*^informal	**0.091** ^***^	**0.145** ^***^	**0.067** ^***^	**3.96** ^**^	0.047
Unemployed^*^formal	0.002	0.001	0.014	0.02	0.887
Unemployed for 0–2 years^*^informal	**0.081** ^***^	**0.010** ^***^	**0.066** ^**^	0.30	0.586
Unemployed for 3–5 years^*^informal	**0.098** ^***^	**0.129** ^**^	0.094^*^	0.12	0.734
Unemployed for 6–8 years^*^informal	**0.165** ^***^	**0.263** ^***^	0.073	1.01	0.314
Unemployed for 0–2 years^*^formal	−0.108	0.018	**−0.230** ^**^	2.47	0.116
Unemployed for 3–5 years^*^formal	0.198^*^	0.289	0.201	0.14	0.704
Unemployed for 6–8 years^*^formal	−0.586^*^	**−1.062** ^***^	−0.113	**47.40** ^***^	0.000

The asterisk symbols following coefficients indicate different levels of statistical significance: ^***^p < 0.01, ^**^p < 0.05, ^*^p < 0.1. Bold values in regression indicate statistically significant coefficients (*P* value < 0.05).

### 3.3 Robust tests

#### 3.3.1 Substitution of moderating variables

In order to test the robustness of above results, this paper constructs a social activity index. The specific construction methods of informal social activity index and formal social activity index are shown in Equation (5) and Equation (6)


(5) $$\begin{array}{l} formal\;index=\overset{4}{\underset{i=1}{\sum }}formal_{i}\;*\;frequency_{i} \end{array}$$

(6) $$\begin{array}{l} informal\;index\;=\overset{3}{\underset{i=1}{\sum }}informal_{i}\;*\;frequency_{i} \end{array}$$

In Equation (5), *formal index* represents the level of participation in formal social activities. The variable *formal*_*i*_ is a binary indicator of whether the respondent engaged in the *i*-th formal social activity (*i* = 1, 2, 3, 4). *frequency*_*i*_ denotes the frequency of participation: 0 for “none,” 1 for “occasionally,” 2 for “weekly,” and 3 for “daily.” This index captures the intensity of formal social participation. In Equation (6), *informal index* represents the degree of involvement in informal social activities. *informal*_*i*_ is a dummy variable indicating participation in the *i*-th informal social activity (*i* = 1, 2, 3), and *frequency*_*i*_ follows the same scoring scheme as above. These indices replace the previous binary indicator for social participation, enabling a more nuanced analysis of its moderating role.

As shown in Tables 7, 8, both the regression results and coefficient comparisons are consistent with previous findings. The negative impact of unemployment on health, particularly among males, remains robust across different model specifications and classification schemes, providing initial evidence for the stability of the main effect model.

**Table 7:** Robust test 1- Main effect of unemployment on health.

**Variables**	**Main effect: Two-class**			**Main effect: Four-class**		
	**Total**	**Male**	**Female**	**Total**	**Male**	**Female**
Unemployed	–0.114^***^	–0.185^***^	–0.082^***^			
	(0.011)	(0.018)	(0.014)			
Unemployed for 0–2 years				–0.143^***^	–0.194^***^	–0.113^***^
				(0.016)	(0.026)	(0.021)
Unemployed for 3–5 years				–0.176^***^	–0.231^***^	–0.133^***^
				(0.030)	(0.054)	(0.038)
Unemployed for 6–8 years				–0.175^***^	**−0.328** ^***^	−0.083
				(0.052)	(0.087)	(0.067)
Informal index	**0.014** ^***^	**0.014** ^***^	**0.015** ^***^	**0.008** ^**^	0.004	**0.011** ^**^
	(0.002)	(0.003)	(0.003)	(0.003)	(0.004)	(0.004)
Formal index	0.007	0.014	−0.002	−0.002	0.018	−0.030^*^
	(0.008)	(0.009)	(0.013)	(0.010)	(0.011)	(0.016)
Observations	18,716	8,588	10,128	11,739	5,675	6,064
R-squared	0.047	0.049	0.053	0.078	0.064	0.097
Respondents	6,264	2,840	3,424	5,181	2,429	2,752

The asterisk symbols following coefficients indicate different levels of statistical significance: ^***^p < 0.01, ^**^p < 0.05, ^*^p < 0.1. Bold values in regression indicate statistically significant coefficients (*P* value < 0.05).

**Table 8:** Robust test 1- Gender comparison of unemployment's effect on health.

**Main effect**	**Total**	**Male**	**Female**	**Test statistics (χ^2^)**	***p*-Value**
Unemployed	**−0.114** ^***^	**−0.185** ^***^	**−0.082** ^***^	**7.25** ^***^	0.007
Unemployed for 0–2 years	**−0.143** ^***^	**−0.194** ^***^	**−0.113** ^***^	1.80	0.179
Unemployed for 3–5 years	**−0.176** ^***^	**−0.231** ^***^	**−0.133** ^***^	0.66	0.416
Unemployed for 6–8 years	**−0.175** ^***^	**−0.328** ^***^	−0.083	0.98	0.323

^***^p < 0.01 (statistically significant at the 1% level). Bold values in regression indicate statistically significant coefficients (*P* value < 0.05).

It can be seen from Tables 9, 10 that in the moderating effect model, the informal social activity index can reduce the average health damage of unemployment. Formal social activity can't reduce the average health damage of unemployment. The buffering effect of the informal social activity index on the health costs of unemployment was significantly greater in men than in women. In the Four-class model, the health damage of 0–2 years of unemployment, 3–5 years of unemployment and 6–8 years of unemployment on men can be significantly buffered by the informal social activity index. For men, the negative effects of 6–8 years of unemployment on health are amplified by formal social participation. For women, the informal social activity index moderates the health damage of unemployment for 0–2 years or 3–5 years, and the negative health effects of unemployment for 0–2 years are amplified by the formal social participation index. In Table 10, the results of the comparison between the moderating coefficients are consistent with those obtained in the previous analysis. Therefore, these results preliminarily indicate that the moderating effect results of this study are robust.

**Table 9:** Robust test 1- Moderating effect of social participation.

**Variables**	**Moderating effect: Two-class**			**Moderating effect: Four-class**		
	**Total**	**Male**	**Female**	**Total**	**Male**	**Female**
Unemployed	**−0.149** ^***^	**−0.217** ^***^	**−0.110** ^***^			
	(0.011)	(0.016)	(0.014)			
Unemployed for 0–2 years				**−0.163** ^***^	**−0.210** ^***^	**−0.130** ^***^
				(0.016)	(0.024)	(0.022)
Unemployed for 3–5 years				**−0.199** ^***^	**−0.210** ^***^	**−0.181** ^***^
				(0.027)	(0.039)	(0.037)
Unemployed for 6–8 years				**−0.277** ^***^	**−0.449** ^***^	**−0.127** ^**^
				(0.042)	(0.059)	(0.060)
Unemployed^*^informal index	**0.024** ^***^	**0.039** ^***^	**0.015** ^***^			
	(0.004)	(0.007)	(0.006)			
Unemployed^*^formal index	−0.013	−0.0001	−0.008			
	(0.018)	(0.030)	(0.024)			
Unemployed for 0–2 years^*^informal index				**0.028** ^***^	**0.037** ^***^	**0.022** ^**^
				(0.007)	(0.011)	(0.010)
Unemployed for 3–5 years^*^informal index				**0.033** ^***^	0.033^*^	**0.036** ^**^
				(0.011)	(0.018)	(0.015)
Unemployed for 6–8 years^*^informal index				**0.042** ^**^	**0.077** ^***^	0.015
				(0.018)	(0.027)	(0.025)
Unemployed for 0–2 years^*^formal index				−0.067^*^	0.029	**−0.133** ^***^
				(0.035)	(0.049)	(0.051)
Unemployed for 3–5 years^*^formal index				0.041	0.119	0.049
				(0.049)	(0.107)	(0.059)
Unemployed for 6–8 years^*^formal index				**−0.642** ^**^	**−1.059** ^***^	−0.211
				(0.300)	(0.392)	(0.455)
Informal index	**0.010** ^***^	**0.007** ^**^	**0.013** ^***^	0.004	0.0004	0.008^*^
	(0.002)	(0.003)	(0.003)	(0.003)	(0.004)	(0.005)
Formal index	0.007	0.011	−0.0005	−0.005	0.006	−0.023
	(0.008)	(0.009)	(0.014)	(0.009)	(0.011)	(0.017)
Observations	25,276	11,730	13,546	15,960	7,930	8,030
R-squared	0.069	0.064	0.077	0.096	0.087	0.113
Respondents	7,577	3,428	4,149	6,548	3,115	3,433

The asterisk symbols following coefficients indicate different levels of statistical significance: ^***^p < 0.01, ^**^p < 0.05, ^*^p < 0.1. Bold values in regression indicate statistically significant coefficients (*P* value < 0.05).

**Table 10:** Robust test 1-Gender comparison of moderating effect of social participation.

**Moderating effect**	**Total**	**Male**	**Female**	**Comparison**	
	**Coefficients**	**Coefficients**	**Coefficients**	**Test statistics (**χ^**2**^**)**	***p*** **-Value**
Unemployed^*^informal index	**0.024** ^***^	**0.039** ^***^	**0.015** ^***^	**4.94** ^**^	0.026
Unemployed^*^formal index	−0.013	−0.0001	−0.008	0.04	0.834
Unemployed for 0–2 years^*^informal index	**0.028** ^***^	**0.037** ^***^	**0.022** ^**^	0.70	0.403
Unemployed for 3–5 years^*^informal index	**0.033** ^***^	0.033^*^	**0.036** ^**^	0.01	0.921
Unemployed for 6–8 years^*^informal index	**0.042** ^**^	**0.077** ^***^	0.015	1.29	0.256
Unemployed for 0–2 years^*^formal index	−0.067^*^	0.029	**−0.133** ^***^	**5.69** ^**^	0.017
Unemployed for 3–5 years^*^formal index	0.041	0.119	0.049	0.34	0.561
Unemployed for 6–8 years^*^formal index	**−0.642** ^**^	**−1.059** ^***^	−0.211	**46.02** ^***^	0.000

The asterisk symbols following coefficients indicate different levels of statistical significance: ^***^p < 0.01, ^**^p < 0.05, ^*^p < 0.1. Bold values in regression indicate statistically significant coefficients (*P* value < 0.05).

Taken together, the results from Tables 7–10 preliminarily indicate that the moderating effects observed in our study are robust when the dummy variable for social participation is replaced with a more nuanced index of participation intensity.

#### 3.3.2 Substitution of samples

In our primary analysis, we excluded respondents who were retired and included people < 80 years old due to that the majority of our rural sample continues working past the typical retirement age. But the effects of unemployment might differ before and after retirement. To test the robustness of our findings, we conducted a separate analysis using 65 years as the age cutoff, thereby focusing exclusively on the effects of unemployment among individuals below 65.

As shown in Tables 11, 12, for men, the negative effects of unemployment on health will increase with the increase of unemployment duration. Consistent with the previous results, this phenomenon is not present in women, and women who have been unemployed for 6–8 years have worse health than women who have not experienced unemployment, but it is not significant. In addition, the average damage to men's health from losing a job was greater than that to women's, but the difference in the impact of unemployment on men's and women's health was not significant in terms of different length of unemployment. In general, the results of main effect regression are robust.

**Table 11:** Robust test 2- Main effect of unemployment on health.

**Variables**	**Main effect: Two-class**			**Main effect: Four-class**		
	**Total**	**Male**	**Female**	**Total**	**Male**	**Female**
Unemployed	**−0.113** ^***^	**−0.185** ^***^	**−0.082** ^***^			
	(0.011)	(0.018)	(0.014)			
Unemployed for 0–2 years				**−0.143** ^***^	**−0.194** ^***^	**−0.114** ^***^
				(0.016)	(0.026)	(0.021)
Unemployed for 3–5 years				**−0.176** ^***^	**−0.229** ^***^	**−0.133** ^***^
				(0.030)	(0.054)	(0.038)
Unemployed for 6–8 years				**−0.175** ^***^	**−0.327** ^***^	−0.084
				(0.052)	(0.087)	(0.067)
Informal	**0.031** ^***^	**0.036** ^***^	**0.028** ^***^	**0.022** ^**^	0.019	0.025^*^
	(0.007)	(0.010)	(0.010)	(0.010)	(0.013)	(0.015)
Formal	0.002	0.006	−0.005	−0.012	0.023	−0.063^*^
	(0.016)	(0.019)	(0.026)	(0.019)	(0.022)	(0.034)
Observations	18,716	8,588	10,128	11,739	5,675	6,064
R-squared	0.046	0.047	0.051	0.078	0.064	0.096
Respondents	6,264	2,840	3,424	5,181	2,429	2,752

The asterisk symbols following coefficients indicate different levels of statistical significance: ^***^p < 0.01, ^**^p < 0.05, ^*^p < 0.1. Bold values in regression indicate statistically significant coefficients (*P* value < 0.05).

**Table 12:** Robust test 2- Gender comparison of unemployment's effect on health.

**Main effect**	**Total**	**Male**	**Female**	**Test statistics (χ^2^)**	***p*-Value**
Unemployed	**−0.113** ^***^	**−0.185** ^***^	**−0.082** ^***^	**7.23** ^***^	0.007
Unemployed for 0–2 years	**−0.143** ^***^	**−0.194** ^***^	**−0.114** ^***^	1.77	0.183
Unemployed for 3–5 years	**−0.176** ^***^	**−0.229** ^***^	**−0.133** ^***^	0.65	0.422
Unemployed for 6–8 years	**−0.175** ^***^	**−0.327** ^***^	−0.084	0.97	0.324

^***^p < 0.01 (statistically significant at the 1% level). Bold values in regression indicate statistically significant coefficients (*P* value < 0.05).

Tables 13, 14 shows the robustness test of the moderating effect by changing samples. Since there are no respondents in the sample aged 65 and below who have been unemployed for 6–8 years and participated in informal social activities, Therefore, the study was unable to capture the buffering effect of formal social participation on the health damage of unemployment for 6–8 years. Overall, the results from the substitution of samples confirm that both the main effects and the moderating effects of social participation on the relationship between unemployment and health are robust, regardless of the sample restrictions.

**Table 13:** Robust test 2- Moderating effect of social participation.

**Variables**	**Moderating effect: Two-class**			**Moderating effect: Four-class**		
	**Total**	**Male**	**Female**	**Total**	**Male**	**Female**
Unemployed	**−0.150** ^***^	**−0.263** ^***^	**−0.105** ^***^			
	(0.014)	(0.024)	(0.018)			
Unemployed for 0–2 years				**−0.191** ^***^	**−0.269** ^***^	**−0.150** ^***^
				(0.021)	(0.035)	(0.028)
Unemployed for 3–5 years				**−0.235** ^***^	**−0.301** ^***^	**−0.190** ^***^
				(0.040)	(0.064)	(0.052)
Unemployed for 6–8 years				**−0.258** ^***^	**−0.549** ^***^	−0.117
				(0.068)	(0.117)	(0.087)
Unemployed^*^informal	**0.076** ^***^	**0.161** ^***^	**0.048** ^**^			
	(0.018)	(0.031)	(0.023)			
Unemployed^*^formal	−0.041	−0.053	−0.009			
	(0.047)	(0.070)	(0.065)			
Unemployed for 0–2 years^*^informal				**0.115** ^***^	**0.161** ^***^	**0.096** ^**^
				(0.030)	(0.048)	(0.039)
Unemployed for 3–5 years^*^informal				**0.110** ^**^	**0.211** ^**^	0.088
				(0.053)	(0.102)	(0.066)
Unemployed for 6–8 years^*^informal				**0.193** ^**^	**0.463** ^***^	0.093
				(0.094)	(0.159)	(0.121)
Unemployed for 0–2 years^*^formal				−0.156^*^	0.014	**−0.361** ^***^
				(0.083)	(0.104)	(0.134)
Unemployed for 3–5 years^*^formal				0.219	0.001	**0.326** ^**^
				(0.136)	(0.272)	(0.166)
Informal	**0.018** ^**^	**0.022** ^**^	0.017	0.008	0.006	0.013
	(0.008)	(0.011)	(0.012)	(0.010)	(0.013)	(0.016)
Formal	0.006	0.009	−0.004	−0.008	0.022	−0.060^*^
	(0.017)	(0.020)	(0.029)	(0.020)	(0.023)	(0.035)
Observations	18,716	8,588	10,128	11,739	5,675	6,064
R-squared	0.047	0.052	0.052	0.081	0.070	0.100
Respondents	6,264	2,840	3,424	5,181	2,429	2,752

The asterisk symbols following coefficients indicate different levels of statistical significance: ^***^p < 0.01, ^**^p < 0.05, ^*^p < 0.1. Bold values in regression indicate statistically significant coefficients (*P* value < 0.05).

**Table 14:** Robust test- Gender comparison of moderating effect of social participation.

**Moderating effect**	**Total**	**Male**	**Female**	**Comparison**	
	**Coefficients**	**Coefficients**	**Coefficients**	**Test statistics (**χ^**2**^**)**	***p*** **-Value**
Unemployed^*^informal	**0.076** ^***^	**0.161** ^***^	**0.048** ^**^	**4.08** ^**^	0.043
Unemployed^*^formal	−0.041	−0.053	−0.009	0.16	0.692
Unemployed for 0–2 years^*^informal	**0.115** ^***^	**0.161** ^***^	**0.096** ^**^	0.53	0.466
Unemployed for 3–5 years^*^informal	**0.110** ^**^	**0.211** ^**^	0.088	0.62	0.432
Unemployed for 6–8 years^*^informal	**0.193** ^**^	**0.463** ^***^	0.093	0.93	0.335
Unemployed for 0–2 years^*^formal	−0.156^*^	0.014	–0.361^***^	3.17^*^	0.075
Unemployed for 3–5 years^*^formal	0.219	0.001	**0.326** ^**^	1.74	0.187
Unemployed for 6–8 years^*^formal	/	/	/	/	/

The asterisk symbols following coefficients indicate different levels of statistical significance: ^***^p < 0.01, ^**^p < 0.05, ^*^p < 0.1. Bold values in regression indicate statistically significant coefficients (*P* value < 0.05).

## 4 Discussion and limitations

### 4.1 Discussion

This study demonstrates that unemployment adversely affects the health of middle-aged and older adults, with the average decline in health being more pronounced among men than women. For middle-aged and older men, the longer the duration of unemployment, the greater the detrimental impact on health. This finding is consistent with (30), who used mortality risk as an indicator of health who used mortality risk as an indicator of health. Under traditional social role constraints, unemployed men are more likely to experience societal stigma in the short term (31). Furthermore, unemployment imposes greater financial pressure on men (26), as unemployment persists, depleting savings and accumulating stress exacerbate health deterioration (32). Thus, with the increase of unemployment duration, men will suffer more and more damage to their health. For middle-aged and older women, unemployment for 0–2 years and 3–5 years will bring health damage, while unemployment for 6–8 years will not cause significant health damage, which is not consistent to previous studies (33). This unexpected pattern may reflect gender differences in coping strategies: long-term unemployed men tend to adopt riskier lifestyles (18), whereas women demonstrate more adaptive coping behaviors (36). Therefore, women's health tends to flatten out when they are unemployed for 6–8 years.

Moreover, the study finds that informal social participation buffers the negative health impacts of unemployment for both genders, with a notably stronger moderating effect among men. In contrast, formal social participation not only fails to mitigate these negative health effects but, under certain conditions, may even exacerbate them. In particular, informal social participation reduces the health costs associated with unemployment across short-, medium-, and long-term periods for men, whereas for women, only short-term unemployment benefits significantly from such buffering. Formal social participation appears to amplify health risks among unemployed middle-aged and older adults—intensifying the negative effects of short-term unemployment among women and long-term unemployment among men.

Regarding the role of formal social participation in the relationship between unemployment and health, this study gives the following explanation: First, formal social activities generally involve lower frequency of participation and smaller groups. According to activity theory, such activities fail to provide continuous, stable role support for individuals coping with the loss of their work role (24). Without a reliable alternative role, formal social participation offers little protection against the health risks triggered by job loss. Second, participation in formal activities can lead to a stratified social comparison process (34), whereby older adults are ranked according to various dimensions. Individuals who suffer significant role losses may perceive themselves as being at the lower end of the hierarchy, thereby intensifying the negative impact of unemployment on health. This hierarchical comparison is primarily evident in secondary relationships—such as those established through formal social participation (35). In addition, the number of people participating in formal social activities is small, so when testing the moderating effect of formal social participation, the coefficient may not be reliable, and the estimate of this coefficient should be treated with caution.

### 4.2 Limitations

Firstly, upon screening the CHARLS questionnaire, only seven types of social participation were identified. Although these seven types encompass the majority of participation behaviors exhibited by middle-aged and older adults, there are still notable omissions. For example, participation in religious activities is an important form of social participation globally. While scholars in Western countries have extensively examined the health impacts of religious participation, similar research in China is sparse. In addition, CHARLS has also ignored online social participation, which is increasingly popular among middle-aged and older adults in the new media era. Future studies should delve deeper into the diversity of social participation in greater depth and further investigate its effects on health.

Secondly, CHARLS inquired about respondents' participation in specific social activities over the past month. However, this approach fails to capture individuals who have regularly engaged in social activities for an extended period but have recently been unable to participate due to illness. To address this limitation, future research should adopt a more comprehensive approach to capture long-term patterns of social activity participation, thereby providing a more accurate representation of individuals' social participation behaviors. Furthermore, although social participation in this study refers to activities in the past month and health is measured as the current status, which helps establish some degree of temporal ordering, we acknowledge that the possibility of reverse causation cannot be entirely ruled out. In future research, we plan to explore suitable instrumental variables to better address this issue.

Thirdly, only respondents' employment status and retirement status are observable in CHARLS, the exact length of unemployment cannot be directly measured. This study defines unemployment duration as a four-category variable based on respondents' employment status across four survey waves. To avoid interference from individuals who have never worked due to various factors, only those who were employed in the first wave are retained. This approach helps ensure that the sample includes individuals who have recently or in the past few years lost their jobs. However, recording job loss by year may overlook respondents who experienced brief reemployment between survey waves. Therefore, future research should employ more specialized survey instruments that capture the continuous duration of unemployment, allowing for a more detailed examination of the relationship between job loss and health.

Fourthly, our final analytic sample accounts for only about 35% of the total initial CHARLS sample, as most attrition was due to partial missing data or participants' unwillingness to complete the lengthy questionnaire, rather than complete dropout caused by health deterioration. Therefore, we believe that the impact of sample attrition on our main results is likely limited. However, we acknowledge that some bias may still exist. Such bias could lead to an underestimation of the true health impact of long-term unemployment, as the least healthy individuals are less likely to be retained in the sample. To address this issue more rigorously in future research, our team plans to design and implement our own survey with tighter control over sample retention and attrition-related biases.

## 5 Conclusion

This study investigates the adverse effects of unemployment on the health of middle-aged and older adults, examines how the duration of unemployment influences these effects, and assesses the moderating role of social participation-particularly informal social activities—with an emphasis on gender differences. The findings indicate that unemployment is detrimental to the health of both middle-aged and older men and women. However, the average health impact is greater for men. For men, the longer the unemployment period, the more severe the health consequences.

In terms of moderating effects, informal social participation plays a protective role against the health costs of unemployment for both genders, while formal social participation does not. In fact, formal participation may even aggravate health outcomes for unemployed individuals. When unemployment duration is considered, informal participation helps mitigate the health impact of short-, medium-, and long-term unemployment among men, but only benefits women during short-term unemployment.

These findings highlight the importance of fostering informal social support networks to alleviate the health consequences of unemployment among older populations, and call into question the assumption that all forms of social participation are inherently beneficial.

## Data Availability

Publicly available datasets were analyzed in this study. This data can be found here: https://charls.charlsdata.com/pages/data/111/zh-cn.html.
