# Supplementary material for: Cumulative effects of unemployment on health in midlife: the buffering effect of social participation
Source: Front Public Health. 2025 Aug 12;13:1611358. doi: 10.3389/fpubh.2025.1611358 (PMC12378039; doi:10.3389/fpubh.2025.1611358)
Supplement: Supplementary file 1 [file Table_1.docx]

**Appendix**

Table A1. Main effect of unemployment on physical health

|  | Main effect: Two-class | | | Main effect：Four-class | | |
| --- | --- | --- | --- | --- | --- | --- |
| Variables | Total | Male | Female | Total | Male | Female |
| Unemployed | **-0.186***** | **-0.275***** | **-0.133***** |  |  |  |
|  | (0.0166) | (0.0254) | (0.0220) |  |  |  |
| Unemployed for 0-2 years | |  |  | **-0.188***** | **-0.236***** | **-0.149***** |
|  |  |  |  | (0.0247) | (0.0361) | (0.0344) |
| Unemployed for 3-5 years | |  |  | **-0.259***** | **-0.280***** | **-0.219***** |
|  |  |  |  | (0.0415) | (0.0624) | (0.0569) |
| Unemployed for 6-8 years | |  |  | **-0.410***** | **-0.705***** | -0.168* |
|  |  |  |  | (0.0659) | (0.0942) | (0.0932) |
| Informal | **0.0326***** | **0.0390**** | 0.0286* | 0.0276* | 0.0174 | 0.0380 |
|  | (0.0120) | (0.0165) | (0.0172) | (0.0159) | (0.0208) | (0.0242) |
| Formal | -0.0233 | -0.0322 | -0.0127 | -0.0570* | -0.0529 | -0.0543 |
|  | (0.0273) | (0.0332) | (0.0444) | (0.0334) | (0.0383) | (0.0597) |
| Observations | 25,276 | 11,730 | 13,546 | 15,960 | 7,930 | 8,030 |
| R-squared | 0.069 | 0.062 | 0.080 | 0.084 | 0.076 | 0.097 |
| Respondents | 7,577 | 3,428 | 4,149 | 6,548 | 3,115 | 3,433 |

Note: All these tables controls for age, income (logarithm), number of chronic diseases, whether they live in the city, marital status, whether their parents are alive, number of living siblings, number of living children, whether they live with their children, whether they have experienced child bereavement, whether they have private insurance, income, whether they smoke, whether they drink, and number of chronic diseases. The standard deviation in parentheses. *** *p*<0.01, ** *p*<0.05, * *p*<0.1.

Table A2. Main effect of unemployment on cognitive health

|  | Main effect: Two-class | | | Main effect：Four-class | | |
| --- | --- | --- | --- | --- | --- | --- |
| Variables | Total | Male | Female | Total | Male | Female |
| Unemployed | **-0.0390**** | **-0.0511**** | -0.0324* |  |  |  |
|  | (0.0153) | (0.0252) | (0.0192) |  |  |  |
| Unemployed for 0-2 years | |  |  | -0.0455* | -0.0618 | -0.0379 |
|  |  |  |  | (0.0239) | (0.0378) | (0.0312) |
| Unemployed for 3-5 years | |  |  | -0.0513 | -0.102 | -0.0240 |
|  |  |  |  | (0.0401) | (0.0653) | (0.0515) |
| Unemployed for 6-8 years | |  |  | -0.0133 | 0.0473 | -0.0501 |
|  |  |  |  | (0.0638) | (0.0987) | (0.0843) |
| Informal | **0.0727***** | **0.0675***** | **0.0793***** | **0.0472***** | **0.0467**** | **0.0502**** |
|  | (0.0111) | (0.0164) | (0.0150) | (0.0154) | (0.0218) | (0.0219) |
| Formal | 0.0407 | 0.0174 | 0.0732* | 0.0226 | 0.0270 | 0.0160 |
|  | (0.0251) | (0.0329) | (0.0388) | (0.0323) | (0.0402) | (0.0540) |
| Observations | 25,276 | 11,730 | 13,546 | 15,960 | 7,930 | 8,030 |
| R-squared | 0.025 | 0.025 | 0.029 | 0.017 | 0.017 | 0.022 |
| Respondents | 7,577 | 3,428 | 4,149 | 6,548 | 3,115 | 3,433 |

Table A3. Main effect of unemployment on psychological health

|  | Main effect: Two-class | | | Main effect：Four-class | | |
| --- | --- | --- | --- | --- | --- | --- |
| Variables | Total | Male | Female | Total | Male | Female |
| Unemployed | **-0.0411**** | -0.0305 | -0.0479* |  |  |  |
|  | (0.0187) | (0.0294) | (0.0245) |  |  |  |
| Unemployed for 0-2 years | |  |  | **-0.0924***** | **-0.0968**** | **-0.0879**** |
|  |  |  |  | (0.0289) | (0.0435) | (0.0392) |
| Unemployed for 3-5 years | |  |  | -0.0264 | -0.00350 | -0.0284 |
|  |  |  |  | (0.0484) | (0.0753) | (0.0647) |
| Unemployed for 6-8 years | |  |  | -0.0933 | -0.118 | -0.0816 |
|  |  |  |  | (0.0769) | (0.114) | (0.106) |
| Informal | 0.00904 | 0.00405 | 0.0122 | -0.0117 | -0.0112 | -0.0147 |
|  | (0.0135) | (0.0191) | (0.0191) | (0.0186) | (0.0251) | (0.0275) |
| Formal | 0.00525 | 0.0541 | -0.0643 | -0.00550 | **0.0932**** | **-0.182***** |
|  | (0.0308) | (0.0383) | (0.0495) | (0.0390) | (0.0463) | (0.0679) |
| Observations | 25,276 | 11,730 | 13,546 | 15,960 | 7,930 | 8,030 |
| R-squared | 0.032 | 0.041 | 0.027 | 0.012 | 0.019 | 0.012 |
| Respondents | 7,577 | 3,428 | 4,149 | 6,548 | 3,115 | 3,433 |
